# Design and In Vitro Evaluation of Layer by Layer siRNA Nanovectors Targeting Breast Tumor Initiating Cells

**DOI:** 10.1371/journal.pone.0091986

**Published:** 2014-04-02

**Authors:** Hamsa Jaganathan, Sucharita Mitra, Srimeenakshi Srinivasan, Bhuvanesh Dave, Biana Godin

**Affiliations:** 1 Department of Nanomedicine, Houston Methodist Research Institute, Houston, Texas, United States of America; 2 Cancer Center of Excellence, Houston Methodist Research Institute, Houston, Texas, United States of America; H.Lee Moffitt Cancer Center & Research Institute, United States of America

## Abstract

Efficient therapeutics and early detection has helped to increase breast cancer survival rates over the years. However, the recurrence of breast cancer remains to be a problem and this may be due to the presence of a small population of cells, called tumor initiating cells (TICs). Breast TICs are resistant to drugs, difficult to detect, and exhibit high self-renewal capabilities. In this study, layer by layer (LBL) small interfering RNA (siRNA) nanovectors (SNVs) were designed to target breast TICs. SNVs were fabricated using alternating layers of poly-L-lysine and siRNA molecules on gold (Au) nanoparticle (NP) surfaces. The stability, cell uptake, and release profile for SNVs were examined. In addition, SNVs reduced TIC-related STAT3 expression levels, CD44^+^/CD24^−^/EpCAM^+^ surface marker levels and the number of mammospheres formed compared to the standard transfection agent. The data from this study show, for the first time, that SNVs in LBL assembly effectively delivers STAT3 siRNA and inhibit the growth of breast TICs *in vitro*.

## Introduction

Development of new therapeutics and employment of early detection practices enabled dramatically improved breast cancer survival rates over the years [1]. However, the recurrence of breast cancer remains to be a threat to cancer survivors [2]. Cancer relapse is especially prominent in patients diagnosed with triple negative breast cancer (TNBC), which is a highly proliferative and aggressive sub-type and resistant to clinically available therapies [3], [4]. One possible explanation for cancer recurrence is the presence of a small population of cells, called tumor initiating cells (TICs) [5], [6], [7]. Breast TICs are resistant to conventional chemotherapeutics and are difficult to detect. They remain quiescent for years and exhibit high self- renewal capabilities. As a result, breast TICs have a potential to grow into new tumors years after chemotherapy. There is a crucial need to focus on treating breast TICs along with the regular tumor cells [8], [9].

In the past decade, small interfering RNAs (siRNAs) have demonstrated a promising therapeutic option for multiple diseases. siRNAs selectively target proteins to down regulate and silence important gene expressions in the cytoplasm of cells [8], [9]. Therefore, siRNAs can potentially target and reduce TIC related protein expressions, thereby selectively killing breast TICs [8]. It is generally agreed in the literature that, although siRNAs have tremendous treatment capabilities attributed to their target specificity, efficiency, and chemical stability, the systemic delivery of these molecules is the major roadblock in their clinical translation posing several challenges of degradation, rapid clearance, and lack of penetration and accumulation in tumors [10], [11], [12].

The main delivery method for siRNAs studied *in vitro* is the use of cationic polymers, such as polyethylenimine (PEI) that create polyplexes with siRNA. PEI, however, has exhibited to be cytotoxic and non-degradable [13], [14], [15]. Therefore, researchers are attempting to design effective delivery vehicles for siRNA with important considerations, including (1) high target specificity, (2) superior cellular uptake and endosomal escape, and (3) low cytotoxicity [10], [14]. There are several approaches to design delivery vehicles with these considerations. Specifically, the layer by layer (LBL) technology has proven to aid in many drug delivery applications. The LBL technology allows for effective encapsulation of drugs or molecules using nanometer thick layers of polyelectrolyes. By encapsulation in LBL, the target drug or molecule is protected from degradation in the body. In addition, due to the layer's ability to shed off, the LBL technology allows for controlled release of drugs or molecules to the intended site, which can be timed, based on the system design, such as number/density of the layers and nature of the polymers [16], [17], [18]. Several groups have demonstrated that the LBL approach on nanoparticles (NPs) efficiently delivered and released siRNAs that downregulated either green fluorescent protein (GFP) or luciferase protein expressions [19], [20], [21], [22], [23]. These proof-of-concept studies demonstrate that LBL is a promising technique to deliver siRNAs with NPs and silence protein expressions.

In this study, siRNA nanovectors (SNVs) are constructed using the LBL approach with gold (Au) NPs stabilized by citrate and poly-L-lysine (PLL) to effectively encapsulate and deliver siRNA to breast TICs. AuNPs have low toxicity and allow for feasible surface modifications to tune desirable charge and hydrophilicity properties [10], [24]. In addition, the use of the cationic polypeptide, PLL, is more biocompatible than PEI and can protect siRNA molecules from nucleases, which can be advantageous for cellular delivery and controlled release from the endosomal compartment [22], [25], [26]. SNVs are examined as a potential therapy option, targeting the signal transducer and activation of transcription (STAT) protein, specifically STAT3. This protein is associated with dysregulated processes in tumors including proliferation, angiogenesis, and metastasis [27]. It was reported that high expression levels of STAT3 are present in breast TICs and are associated with TIC self-renewal [28].

Herein, we demonstrate a promising treatment method for breast TICs using SNVs, which utilize LBL technology on AuNPs. First, the formation of SNVs is characterized by zeta potential, hydrodynamic size, and surface plasmon resonance (SPR). Then, the stability, cell uptake, and release of SNVs are examined and the effect on down regulating phosphorylated STAT3 (p-STAT3) expression levels is confirmed by western blot, the reduction of CD44^+^/CD24^−^/EpCAM^+^ fraction in flow cytometry and mammosphere assays.

## Methods

### Materials

Au(III) chloride (>99.99%), poly-L-lysine and poly-L-lysine tagged with fluorescein isothiocyanate (FITC) (MW 70 KDa) were purchased from Sigma-Aldrich Co (St Louis, MO). siRNA molecules were also purchased from Sigma Aldrich Co. (scrambled: GUGCAGUAUCCUCUGACAG and STAT3: CCAAGUUCAUGGCCUUAGGUAG) For cell culture, Dulbecco's Modified Eagle Medium (DMEM), fetal bovine serum (FBS), and penicillin-streptomycin were purchased from Gibco® (Invitrogen Corporation, Carlsbad, CA). The cell line, SUM159, was purchased from Asterand, Inc (Detroit, MI) and MDA-MB-231 and MCF10A were purchased from ATCC (American Tissue Culture Collection, Manassas, VA). Mammosphere media was made using FGF-Basic (Invitrogen), B-27 serum free supplement (Invitrogen), 0.2% heparin (Stemcell Technologies Inc., Newark, CA) and MEGM growth media (Lonza Inc., Walkersville, MD). siPORT, a polyamine-based transfection agent, was used as positive control for siRNA delivery studies and was purchased from Invitrogen. siRNA labeling kit was purchased from Invitrogen (Ambion® Silencer® siRNA Labeling Kit-Cy3).

### Methods

#### Fabrication of SNVs

AuNPs were fabricated by the citrate-reduction procedure using a mass ratio of 1∶1 sodium citrate∶Au(III) chloride [29]. LBL formation with siRNAs on AuNP systems has been presented in several publications [20], [21], [22], [23], [30], [31], [32]. In this study, PLL solution was added to AuNP solution and mixed for 5 minutes. Then, using the Amicon® Ultra-0.5 mL centrifugal filter system (EMD Millipore, Billerica, MA), the NPs were washed at 2000×g for 2 minutes and retained at 1000×g for 1 minute. The second layer was added with negatively charged siRNA molecules and the mixing and washing steps were repeated. Lastly, the third layer of PLL was added again and mixed and washed.

#### Characterization

Absorbance spectra of SNVs were measured ranging from 400–700 nm using the Synergy H4 Hybrid Multi-mode Microplate Reader (BioTek Instruments, Inc, Winooski, Vermont) with Gen5.1.11 software. Zeta potential and hydrodynamic size were measured using the dynamic light scattering (DLS) instrument (Malvern Zetasizer Nano Series, Worcestershire, UK). Fluorescently labeled siRNA was used to calculate the amount of siRNA molecules encapsulated between the PLL layers of the SNVs. MultiMode® atomic force microscope (Bruker Corporation, Billerica, MA) in tapping mode under standard air conditions was used to image the topography of SNVs.

#### Cell culture

SUM159 and MDA-MDA-231 were cultured in DMEM with 10% FBS and 1% penicillin and streptomycin at 37°C in 5%CO_2_ conditions. MCF10A was cultured in DMEM/F12 media with 5% horse serum, 20 ng/mL epidermal growth factor (EGF, Peprotech), 500 µg/mL hydrocortisone (Sigma), 100 ng/mL cholera toxin (Sigma), 10 µg/mL insulin (Sigma) and 1% penicillin and streptomycin.

#### Toxicity and Release studies

The release of layers from the SNVs was measured using FITC tagged PLL. PLL-FITC was added as the first layer on bare AuNPs stabilized by citrate. Then, siRNA molecules and PLL (without a fluorescent tag) was added as the second and third layer, respectively. The SNVs were then dispersed into FBS (pH 7.2) and deionized water (pH 5.5) at 37°C. At time points ranging between 30 min to 2 days, the amount of PLL-FITC released out of the NP system was measured by fluorescent spectrometer (Ex: 488 nm, Em: 525 nm). Release of siRNA (layer 2) was performed similarly using Cy3 tagged siRNA molecules and read at 550/570 nm Ex/Em. Cell toxicity was evaluated using WST-1 assay on SUM159 and MCF10A cells. After treatment, cells were washed and incubated with 10% WST-1 solution for 2 h. The absorbance was measured at 450 nm using the spectrometer. In order to image the release, cells were seeded on glass slides and PLL-FITC layered AuNPs were added to the cells. After time points (4, 24, and 48 h), the cells were fixed with 4% paraformaldehyde (PFA) and stained with DAPI. They were imaged under the FITC and DAPI filter using the NIKON® fluorescent inverted microscope (Belmont, CA). In addition, LSRFortessa cell analyzer (BD Biosciences, San Jose, CA) was used to quantify the number of cells that internalized SNVs after 4, 24, and 48 h. These SNVs were constructed with PLL-FITC as the last, top layer and were measured with the FITC laser.

#### Western blot analysis

Cells were lysed and assayed for Western blot, testing p-STAT3 protein expression levels after a 72 h treatment with bare AuNPs, non-targeted and STAT3 targeted siRNA molecules in siPORT, and non-targeted and STAT3 targeted SNVs on TNBC cell lines, SUM159 and MDA-MB-231. Briefly, 50 µg of whole cell extracts were quantified and run on a 4–20% gel for 1 h and transferred onto nitrocellulose membranes. The primary antibody (p-STAT) was incubated overnight followed by 1 h secondary antibody incubation, and developed using chemiluminescence.

#### Mammosphere assay

Culturing mammosphere was performed as previously described [33], [34], [35]. In short, single cells dispersed in mammosphere media were plated in 24 well ultra-low attachment plates (Corning Inc., Corning, NY). The cells were plated at a density of 5000 viable cells per well. After 3 days, primary mammospheres were counted using GelCount colony counter (Oxford Optronix, Oxfordshire, UK). Then, primary mammospheres were disassociated and re-seeded at 5000 cells per well in fresh mammosphere media. After 3 days, secondary mammospheres were counted. Mammosphere forming efficiency (MSFE) was calculated as a percentage of mammospheres count from total number of cells seeded (5000 cells).

#### Flow Cytometry

After a 72 h treatment, SUM159 and MCF10A cultures were trypsinized, counted, washed with phosphate-buffered saline (PBS) and stained with cell surface markers: FITC anti-human CD326 (EpCAM) (BioLegend, Inc., San Diego, CA), PE-Cy7 Mouse Anti-Human CD24 (BD Biosciences, San Jose, CA), and APC Mouse Anti-Human CD44 (BD Biosciences, San Jose, CA). One million cells were incubated with the antibodies for 15 minutes in dark on ice with manufacturer recommended concentrations. After washing with PBS, cells were analyzed using the LSRFortessa cell analyzer (BD Biosciences, San Jose, CA). Side and forward scatter were used to eliminate debris and cell doublets. Unstained and IgG2a, κ isotypes with PE-Cy7, FITC, and PE (BD Biosciences, San Jose, CA) stained samples were used as controls.

#### Statistical Analysis

Test for significance between groups was performed using one-way analysis of variance with a confidence level of 95% in STATA 10.0 (StataCorp LP, College Station, TX).

## Results

**Figure 1:**
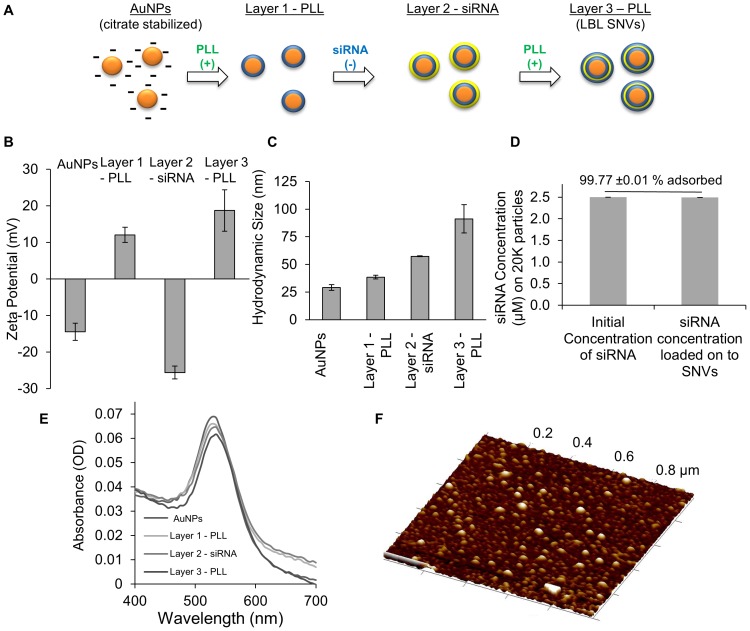
Schematic representation on the fabrication method for SNVs using AuNPs. Plot of (**B**) zeta potential, (**C**) hydrodynamic diameter, (**D**) the amount of siRNA loaded onto SNVs and (**E**) absorbance for bare AuNPs stabilized by citrate and every subsequent layer added on to the SNVs. (**F**) AFM height image of SNVs.

The LBL formation on NP systems has been presented in several publications [36], [37], [38]. SNVs made by the LBL approach on AuNPs to encapsulate siRNAs were formed and characterized. PLL, a positively charged polyelectrolyte, was electrostatically attached onto the surfaces of the AuNPs, stabilized by negatively charged citrate molecules. Then a second layer of negatively charged siRNA molecules was electrostatically attached to positively charged PLL layer. The last layer of PLL was added to encapsulate surface bound siRNA molecules in between two PLL layers to form SNVs (**Figure 1A**).

The physicochemical features of the final SNV design were characterized by zeta potential, hydrodynamic diameter, and surface plasmon resonance (SPR). The addition of each positively charged polyelectrolye - PLL and negatively charged molecules - siRNA on AuNPs surfaces caused the zeta potential to alternate, confirming surface modifications on AuNPs. With PLL as the last layer on SNVs, zeta potential was positive at +18.7±5.7 mV and the final hydrodynamic diameter was 91.3±12.8 nm (**Figure 1B and C**). The amount of siRNA molecules attached on AuNPs was calculated by differences in fluorescent intensity using Cy3-labeled siRNA. It was calculated that the 99.77±0.01% of siRNA molecules added to solution were adsorbed to 20,000 AuNPs (**Figure 1D**). The polydispersity index after each additional layer was below 0.2, suggesting that the SNVs were monodispersed and not aggregated. The addition of the last layer on SNVs caused a slight red shift in the SPR band from 530 nm to 535 nm and a reduction in absorbance by 11% compared to bare AuNPs (**Figure 1E**). Atomic force microscope (AFM) image verified a spherical morphology for SNVs (**Figure 1F**).

**Figure 2:**
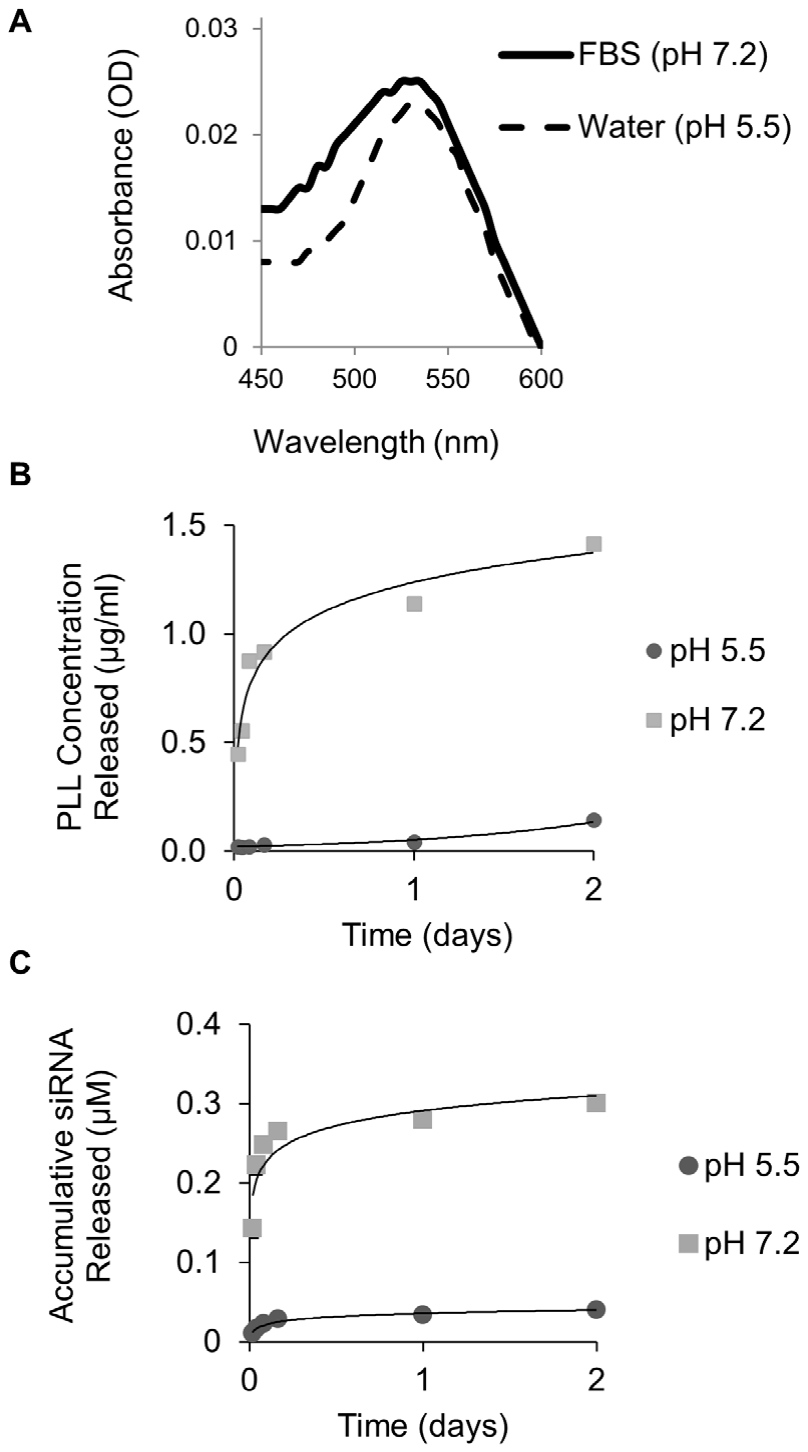
(**A**) Absorption spectra of SNVs in FBS and in water. (**B**) Release profile of the first layer (FITC tagged poly-L-Lysine) released from LBL SNVs in pH 5.5 and 7.2 at 37°C. (**C**) Release profile of the STAT3 siRNA layer (Cy3-tagged siRNA molecules) released from the SNVs in pH 5.5 and pH 7.2 at 37°C.

In order to evaluate stability, SNVs were dispersed in 70% FBS, which contained serum proteins and growth factors. Changes in SPR band, which is influenced by particle size, shape, aggregation state, and surrounding media, were assessed. The SPR band after the addition of the last layer for SNVs in FBS caused the absorbance spectrum to broaden and a slight red band shift when compared to SNVs dispersed in water (**Figure 2A**). The broadened spectrum and the wavelength shift occurred due to the adsorption of serum proteins to SNVs [39].

The release profile of the layers from the SNVs was studied in simulated cytoplasmic (pH 7.2) and endosomal (pH 5.5) pH environments. The release profile of the first layer (PLL-FITC) in pH 5.5 displayed an exponential fit with around 91% of the total concentration attached to AuNPs releasing out in 2 days. On the contrary, around 80% of PLL-FITC total concentration was released out from SNVs into FBS solution within 2 days, fitting to a logarithmic release profile (**Figure 2B**).

The release profile of the siRNA layer (Layer 2) from SNVs was also studied in simulated cytoplasmic (pH 7.2) and endosomal (pH 5.5) pH environments (**Figure 2C**). Although the release of siRNA molecules is limited by the release rate of PLL (Layer 3), siRNA molecules have a faster release profile in the cytoplasm simulated environment than in the endosomal simulated environment. The cytoplasm is a highly reductive environment and therefore, the components from the SNV may undergo redox reactions that can trigger the release of siRNAs from nanoparticle platforms [10], [30].

**Figure 3:**
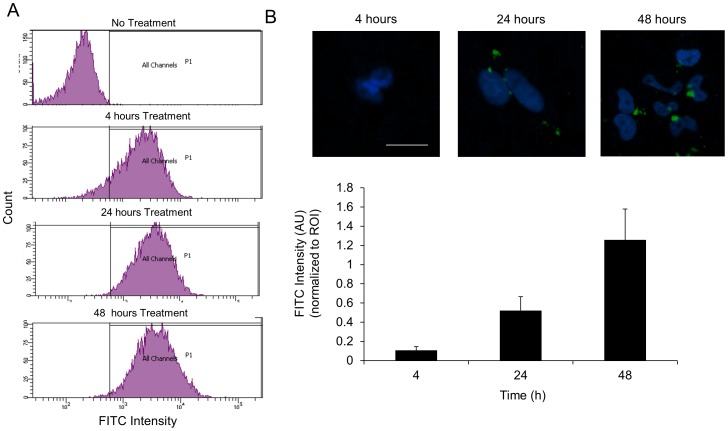
(**A**) Number of cells emitting FITC after uptake of FITC tagged PLL layer (Layer 3-green) from SNVs after 4, 24 and 48 h treatment. (**B**) Plot of the FITC intensity measured at each time point, 4, 24, and 48 h (n = 9, n is number of regions of interest (ROI) from fluorescent images) Images correspond to fluorescent image of SUM159 cells (nuclei stained with DAPI – blue color) displaying the release of FITC tagged PLL layer (Layer 1-green) from SNVs, scale bar = 10 µm.

Following release studies, cell toxicity and uptake studies were performed. After treatment with various NP and siRNA concentrations, cell toxicity was not observed to normal breast cancer cells (MCF10A) and TNBC cells (SUM159) (**Supporting Information Figure S1**). SUM159 cell line expresses many genes related to the breast TIC sub-population [28]. For uptake studies, SNVs were constructed with PLL-FITC as the last, top layer on the surface. Flow cytometry results confirmed that 95% of the cell population was able to internalize SNVs within 4 h and maintained fluorescence (emitted from PLL-FITC) up to 48 h (**Figure 3A**). For release studies, SNVs were constructed with PLL-FITC as the first layer on the surface. Images of SUM159 cells (nuclei stained with DAPI-blue) display the cellular internalization and release in cytoplasm for SNVs (**Figure 3B**). The fluorescent intensity (in green color) emitted by PLL-FITC was observed after 4, 24, and 48 h of treatment. Cells exhibited a low FITC intensity after 24 h, but intensity increased within 48 h, verifying the slow 48 h release of PLL-FITC layer from SNVs. By 48 h, the cytoplasmic area exhibit high fluorescent intensity, indicating that the components from the AuNPs were dispersed in the cell.

After confirming cell internalization and release of layers, the efficiency of gene silencing was examined through western blot and flow cytometry and confirmed by primary and secondary mammosphere formation assays. The mammosphere assay is used to assess the subpopulation of TICs from heterogenic cancer cell lines, such as the TNBC cells, SUM159, under *in vitro* conditions [8]. TICs in a specific serum-free media form mammospheres, which are suspended clusters of breast cancer cells with stem cell-like gene signatures. The number of mammospheres formed is an indicator for the presence of TICs in a cell population. The efficiency of silencing STAT3 expression was compared between SNVs and siRNA encapsulated in siPORT, a polyamine-based transfection agent used as a delivery standard in *in vitro* assays. Additionally, non-targeted (scrambled) and targeted siRNA against STAT3 was compared to test specificity of the resulting SNVs. Flow cytometry was used to examine TIC related surface markers: CD44, CD24, and EpCAM. The overexpression of CD44 and EpCAM with a low expression of CD24 has been related to breast TICs [40].

**Figure 4:**
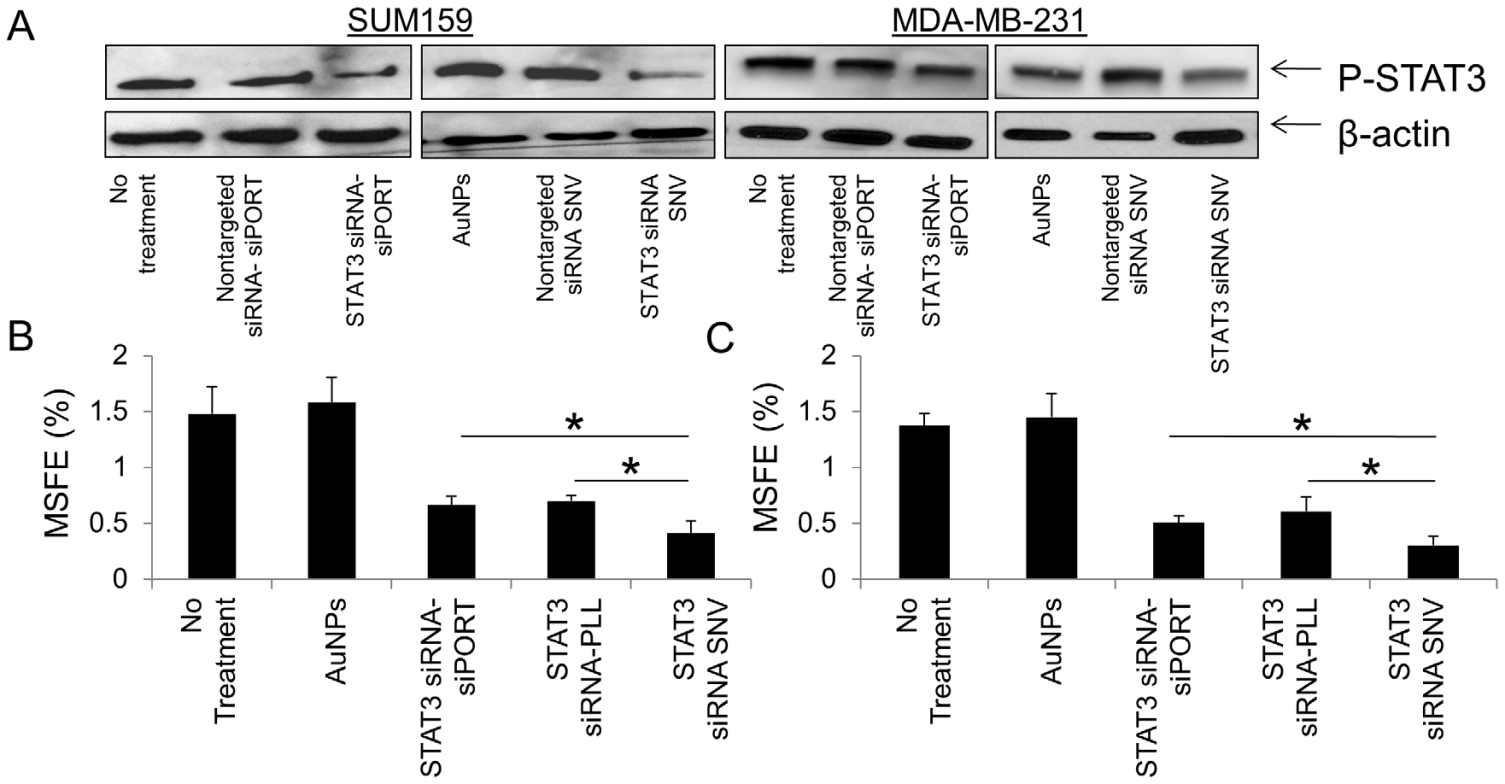
(**A**) Protein expression of p-STAT3 pathway in triple negative breast cancer cell lines, SUM159 and MDA-MB-231, after treatment with AuNPs, non-targeting siRNA in siPORT, STAT3 targeting siRNA in siPORT, non-targeting SNVs, and STAT3 targeting siRNA in SNVs. (**B**) Primary mammosphere forming efficiency from breast cancer cells after 3 days treatment with AuNPs, STAT3 siRNA in siPORT, in PLL and STAT3 targeting siRNA in SNVs. (**C**) Forming efficiency of secondary mammospheres was plotted after primary mammospheres were collected, disassociated, and re-seeded in fresh mammosphere media for 3 days. (* = statistical significance, n = 6, p<0.05).)

The expression levels of p-STAT3 decreased when cells were treated with STAT3 targeting siRNA compared to non-treated cells (**Figure 4A**). With the treatment of STAT3 targeting SNVs, p-STAT3 expression levels were lower than treatment with STAT3 targeting siRNA (encapsulated in siPORT) alone, exhibiting high silencing efficiency. The non-targeted siRNA in SNVs did not affect STAT3 pathway, indicating high specificity from the STAT3 targeted SNVs to breast TICs. Similar results were also observed in another TNBC cell line, MDA-MB-231.

**Figure 4B** displays the plot of primary mammospheres forming efficiency from SUM159 cells after 3 days treatment with bare AuNPs, STAT3 targeting siRNA in siPORT, STAT3 targeting siRNA in PLL complex, and STAT3 targeting SNVs. Primary mammosphere formation was not affected by the treatment of bare AuNPs. After the treatment of STAT3 targeting siRNA molecules in siPORT and STAT3 targeting siRNA in PLL complex, primary mammosphere formation decreased when compared to mammosphere formation from non-treated cells. The formation of primary mammospheres after the treatment from STAT3 targeting SNVs significantly decreased by around 72% compared to mammosphere formation from non-treated cells. The reduced number of primary mammosphere formation is directly related to effective STAT3 silencing in breast TICs by SNVs.

Secondary mammosphere assay, or the second passage from the cells of primary mammospheres, is used to evaluate the self renewal capability of TICs. The formation of secondary mammospheres from cells initially treated with STAT3 targeting SNVs significantly decreased by around 77% compared to secondary mammopsheres formed by non-treated cells and by 40% compared to secondary mammospheres formed from cells treated with STAT3 targeting siRNA in siPORT, and in PLL (**Figure 4C**). Images of mammospheres are shown in **Supporting Information Figure S2**.

**Figure 5:**
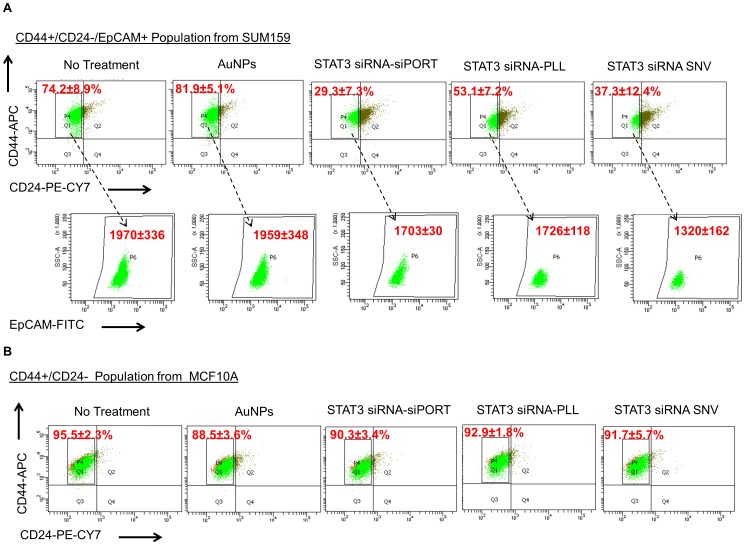
(**A**) CD44^+^/CD24^−^ expression profiles for SUM159 cells treated for 3 days with STAT3 siRNA in either siPORT, PLL, or SNV comparing to no treatment and treatment with AuNPs alone. Values are the % parent population in Q1 that correspond to the CD44^+^/CD24^−^ population. Arrows point to the flow cytometry analysis and gates for EpCAM+ on the CD44^+^/CD24^−^ population for SUM159 cells. Values correspond to the median FITC intensity. (**C**) CD44^+^/CD24^−^ expression profiles for MCF10A cells treated with STAT3 siRNA in either siPORT, PLL, or SNV comparing to no treatment and treatment with AuNPs alone. Values are the % parent population in Q1 that correspond to the CD44^+^/CD24^−^ population.

Furthermore, MCF10A and SUM159 cells were stained for TIC related surface markers, CD44^+^/CD24^−^/EpCAM^+^, to examine the change in TIC expression levels after treatment with STAT3 targeting siRNA in siPORT, in PLL alone, and in SNV configuration. The delivery of STAT3 targeting siRNA to SUM159 cells reduced the fraction of CD44^+^/CD24^−^ population when compared to SUM159 cells without treatment and treatment with bare AuNPs (**Figure 5A**). Additionally, as measured by the reduction in FITC intensity, the expression levels of EpCAM reduced after treatment with STAT3 siRNA for all delivery methods (siPORT, PLL, and SNV), signifying the reduction in breast TICs. The delivery of STAT3 targeting siRNA through SNVs significantly reduced the CD44^+^/CD24^−^/EpCAM+ fraction when compared to non-treated cells and cells treated with STAT3 in PLL and siPORT. Conversely, there was no change in expression levels for CD44^+^/CD24^−^ in MCF10A cells after treatment with SNVs and their components (**Figure 5B**). It was previously shown that MCF10A does not show consistency when stained for CD44+/C24−. As explained by Sheridan et al. [41], the percentage of CD44+/CD24− subpopulation dramatically varied from experiment to experiment and they believe it is due to the serum in the cell cultures. Therefore, although in our study, the CD44+/CD24− sub-population of MCF10A was high in numbers, it may not accurately represent the percentage of normal breast epithelium CD44+/CD24− observed *in vivo*. For future *in vivo* studies, specificity assays will be performed and tested for the changes in STAT3 levels for normal breast tissues due to the SNV treatment. EpCAM was also stained for MCF10A (data not shown), however, the additional staining for EpCAM did not provide any further insight to the data as there were no changes in cell population percentages after the different treatments. The delivery of STAT3 targeting siRNA in siPORT, PLL, or SNV did not affect the expression in normal breast epithelial cells.

## Discussion

Evident from the physicochemical characteristics, the LBL design of SNVs effectively encapsulated siRNA molecules in between PLL layers on the surface of AuNPs. The alternating negative and positive zeta potentials and the reduction in absorbance prove that there was adequate association and coverage among the layers on AuNP surfaces. The additional layers increased the size of the particles, and therefore, decreased the distance between adjacent NPs in solution. This causes an increased overlap of the surface plasmon between adjacent NPs and a red shift in the SPR band for SNVs. Furthermore, the final hydrodynamic size of the SNVs was in between the standard size range of 20 to 200 nm, which aids for efficient passive tumor accumulation through enhanced permeation and retention (EPR) effect [10], [14].

For systemic delivery, SNVs must exhibit stability and a controlled release mechanism of each layer. Under *in vivo* conditions, negatively charged serum opsonins tend to aggregate with positively charged NP surfaces, resulting in rapid clearance from the plasma through the reticulo-endothelial system (RES) [14]. The stability and release of the layers from SNVs were examined in simulated *in vivo* environments with pH 5.5 and 7. Along with exhibiting different release curves, there was a slower release rate for PLL-FITC in acidic pH environment compared to physiological pH environment. The differences in release profiles suggest the pH of an environment is an important factor in the de-construction of the SNV layers. Since SNVs are promptly internalized by cells, this feature of slow release and stabilization of the complexes in the low pH is advantageous in order to control the release and delivery pathway for siRNAs in the cells, from endosomes into cytoplasm [32].

Studies have shown that AuNPs carrying siRNAs in the LBL form internalize in cells by the endocytic pathway [21], [30], [31], [32]. Zhao *et. al.* demonstrated that zeta potential of LBL modified AuNP systems affects the internalization efficiency [30]. For their AuNP systems, zeta potentials at +35 mV produced low cell uptake, whereas, zeta potentials of around +25 mV exhibited optimal cell uptake in human breast cancer cell line, MCF7. Nanoparticle systems with high zeta potentials cause a strong electrostatic attraction to negatively charged cell membranes. The strong attraction may disrupt the membrane structure instead of getting internalized through endocytosis mechanism [30]. In this study, SNVs exhibited a final zeta potential of around 18 mV, which allowed for adequate cell internalization. The addition of more layers on SNVs may increase the zeta potential and be detrimental to the cell uptake behaviour.

The observed high silencing efficiency of STAT3 expression with SNVs is due to the cell uptake from optimal SNV surface conditions, as well as, the stability of siRNA in the cytoplasm. Additionally evident from the CD44^+^/CD24^−^ fraction, the TIC subpopulation significantly reduced for SUM159 after treatment with SNVs. The CD44^+^/CD24^−^/EpCAM^+^ fraction after treatment with SNVs was significantly reduced when compared to the other delivery methods (PLL and siPORT) for STAT3 and to the non-treated cells. The LBL assembly on AuNPs can explain the reduction in CD44^+^/CD24^−^/EpCAM^+^ fraction and responses to mammosphere formation among SNVs, PLL, and siPORT. The LBL assembly on AuNPs protected siRNA stability and aided to maintain high siRNA concentrations in the cytoplasm. Therefore, SNVs showed a greater response in primary and secondary mammosphere formation. siRNA molecules in siPORT and in PLL may have degraded in a faster rate than siRNA in the LBL-Au assembly or may have not completely released out, decreasing the local concentration of siRNA in the cytoplasm and thereby, exhibiting lower mammosphere formation and gene silencing efficiencies when compared to SNVs. Secondary mammosphere formation analysis demonstrates siRNA molecules delivered by SNVs effectively also reduced the capability of breast TIC self renewal.

Specificity of SNVs to TNBCs was evaluated by testing the effect of STAT3 targeting siRNA in SNV configuration on normal breast epithelial cell line (MCF10A). No sign of toxicity for MCF10A and SUM159 was observed from the STAT3 siRNA in the SNV configuration, after treatment with various concentrations of NPs and siRNA. Additionally, STAT3 has been detected mostly in breast cancer cells, but not in normal breast tissues [42]. Therefore, no change in surface markers (CD44 and CD24) was observed after treatment with STAT3 targeting siRNA in different delivery methods to MCF10A cells *in vitro*.

### Conclusions

This study presents a potential therapy to breast TICs. SNVs are shown to exhibit stability, high cell uptake, and effective silencing of STAT3 protein expression in TNBC cells, specifically. These promising results suggest that SNVs can be used in a combinational therapy to treat breast cancer and prevent recurrence by eradicating primary tumors and targeting TICs to reduce their self renewal capability.

## Supporting Information

Figure S1 (**A**) Cell toxicity tested at a constant STAT3 targeting siRNA concentration of 50 nM with various numbers of SNV particles per1000 cells seeded. (**B**) Cell toxicity tested at a constant number of SNV particles (20 K) with various concentrations of STAT3 targeting siRNA treated for 3 days per 1000 cells seeded for MCF10A and SUM159 cell lines. (* = statistical significance between the means, n = 6, p<0.05).
(TIF)

Figure S2 **Images of primary and secondary mammospheres after treatment (scale bar = 200 µm).** Yellow arrow point to the general area of mammospheres and large colonies of mammospheres are circles in yellow.
(TIF)
