# Supplementary figures and images for: Design and In Vitro Evaluation of Layer by Layer siRNA Nanovectors Targeting Breast Tumor Initiating Cells
Source: PLoS One. 2014 Apr 2;9(4):e91986. doi: 10.1371/journal.pone.0091986 (PMC3973666; doi:10.1371/journal.pone.0091986)

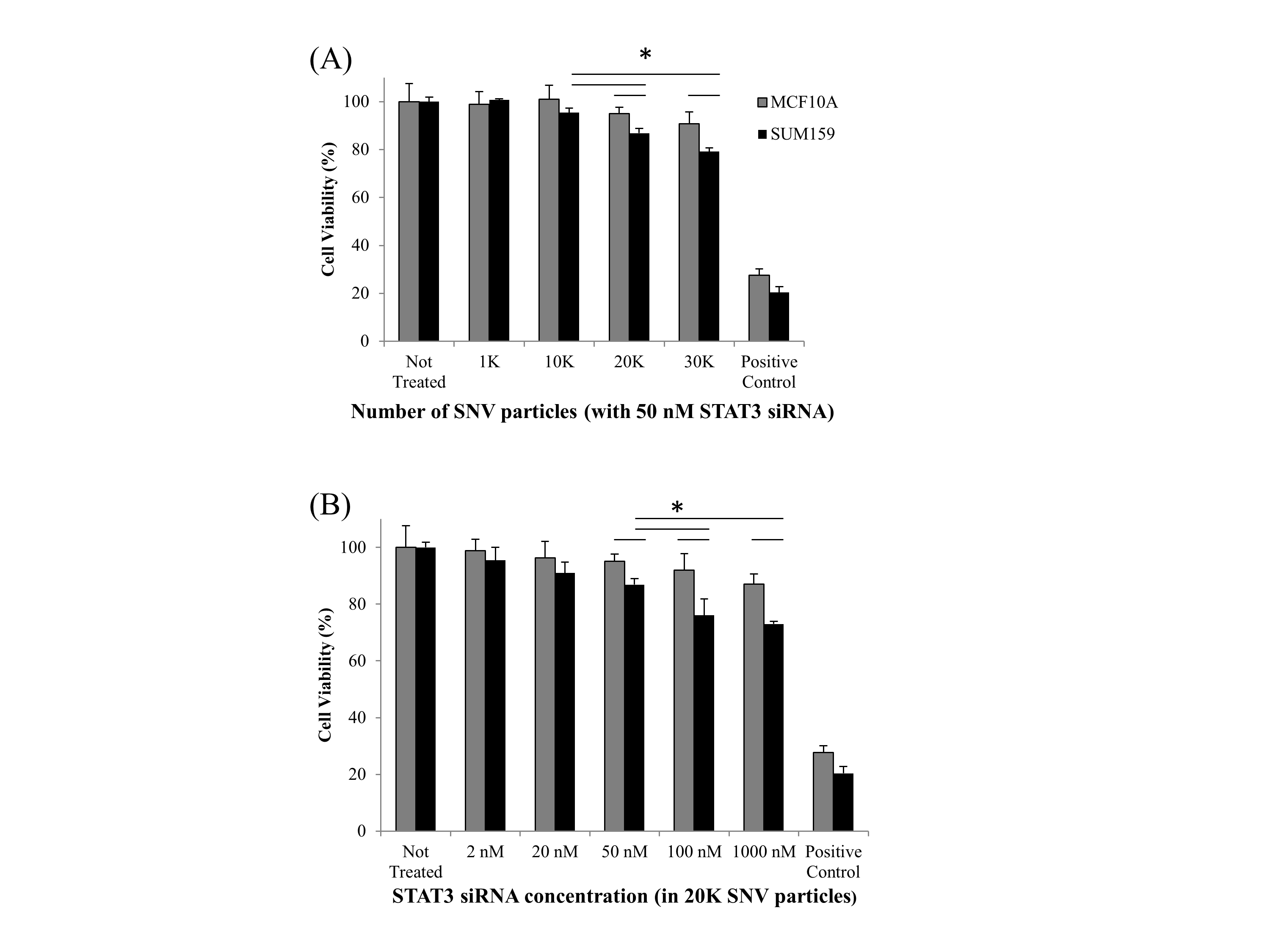

Supplement: Figure S1 — (A) Cell toxicity tested at a constant STAT3 targeting siRNA concentration of 50 nM with various numbers of SNV particles per1000 cells seeded. (B) Cell toxicity tested at a constant number of SNV particles (20 K) with various concentrations of STAT3 targeting siRNA treated for 3 days per 1000 cells seeded for MCF10A and SUM159 cell lines. (* = statistical significance between the means, n = 6, p<0.05). (TIF) [file pone.0091986.s001.tif]

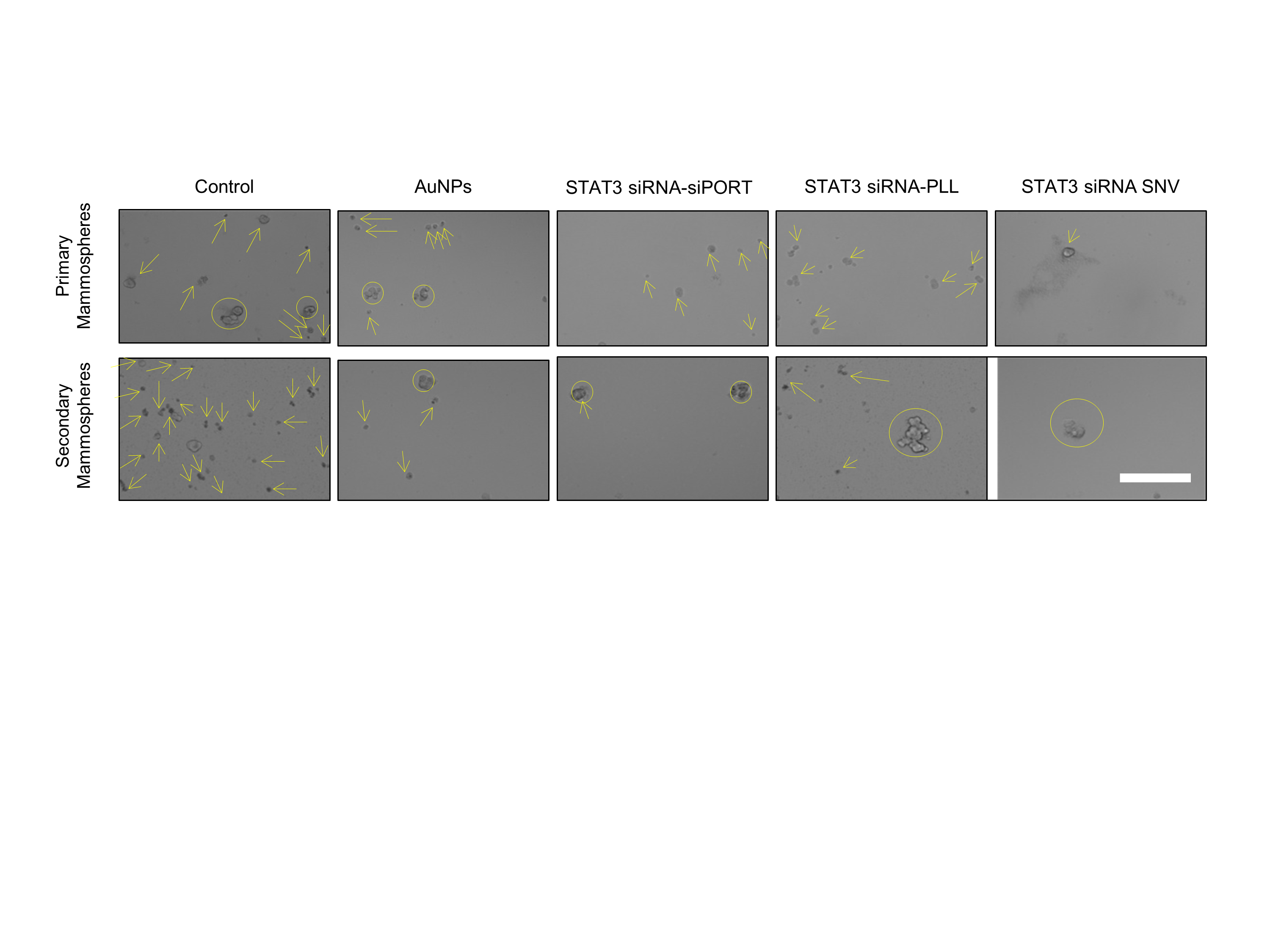

Supplement: Figure S2 — Images of primary and secondary mammospheres after treatment (scale bar = 200 µm). Yellow arrow point to the general area of mammospheres and large colonies of mammospheres are circles in yellow. (TIF) [file pone.0091986.s002.tif]
